# Physiological Characteristics and Environment Adaptability of Reef-Building Corals at the Wuzhizhou Island of South China Sea

**DOI:** 10.3389/fphys.2020.00390

**Published:** 2020-04-29

**Authors:** Huili Xu, Boxuan Feng, Minrui Xie, Yuxiao Ren, Jingquan Xia, Yu Zhang, Aimin Wang, Xiubao Li

**Affiliations:** ^1^State Key Laboratory of Marine Resource Utilization in South China Sea, Hainan University, Haikou, China; ^2^College of Marine Science, Hainan University, Haikou, China

**Keywords:** scleractinian coral, environmental stress, zooxanthella, trade-off, Wuzhizhou Island

## Abstract

The health of coral reef has declined significantly around the world due to the impact of human activities and natural environment changes, and corals have to develop effective resistance mechanisms to survive. In this study, we examined the physiological characteristics and *Symbiodiniaceae* types of four dominant scleractinian corals in the reefs at the Wuzhizhou Island (WZZ) in South China Sea. The water environmental conditions are complex on the north side of WZZ due to regional geography and tourism development, and all corals had their unique physiological conditions and *Symbiodiniaceae* types. For all corals of this study, the rETR_m__ax_ and protein content were significantly lower and the SOD enzyme activity was significantly higher in the north than in the south. Interestingly, ITS2 genotyping showed that *Galaxea fascicularis* contained dominant *Symbiodiniaceae* either genotype C21 or D1a depending on the regional environmental stress, and had stronger heterotrophy than the other three coral species. In addition, the light use efficiency of the dominant *Symbiodiniaceae* type C1 for *Pocillopora verrucosa* was significantly lower in the north and the half saturating irradiance was stable. Besides, *Montipora truncata* and *P. verrucosa* increased their density of the symbiotic zooxanthella C1 in the north to offset the decline of photosynthetic efficiency and thus supply energy. For *Porites lutea* and *G. fascicularis*, their half saturating irradiance declined sharply in the north, where *P. lutea* resorted to heterotrophic feeding to balance the energy budget when the number of zooxanthellas fell short and *G. fascicularis* reduced its energy reserve significantly when the energy source was limited. We thus demonstrated the differences in the physiological responses and energy metabolism strategies between the zooxanthella and the host coral of the four reef-building coral species under the stress of complex water environment on the north side of WZZ. The corals were found to cope with natural and anthropogenic stressors by adjusting the nutrient input sources and the energy structure metabolism of coral hosts or adapting to more sustainable relationship with *Symbiodiniaceae* clades. The corals exhibited their capacity against long-term disturbances by developing their own successful resistance mechanisms at symbiotic relationship and energy metabolism level.

## Introduction

In recent decades, coral reef ecosystems have seriously degraded worldwide due to anthropogenic impact and natural stress (Guest et al., 2016). Degradations may be triggered by a series of incidents, including coral bleaching, sewage discharge from coastal development, eutrophication, disease outbreak, widespread failure of larva supplements, etc. (Hughes et al., 2010). Coral bleaching results from the physical and symbiotic changes in the algae and the stress related to the host, and involves the symbiotic relationship between corals and zooxanthellae (Fitt et al., 2000). After bleaching events, different species of corals may recover or adapt differently. During the 1998 Maldivian bleaching event, the dominant species (e.g.: Acroporidae and Pocilloporidae) were practically disappeared and Poritidae survived partly, while in the subsequent recovery, Agariciidae (esp. Pavona) became a new dominant community and regenerations of Porites increased obviously (Loch et al., 2002). In the coral reefs of Panama, there was a significant increase in the number of corals symbiotic with clade D zooxanthellae, which increased from 43% in 1995 to 63% in 2001 (Baker et al., 2004). Thus, corals may continue to survive if they can withstand the impacts of environment changes.

Environmental stress (e.g., typhoon, high temperature, strong ultraviolet radiation, increased turbidity, and higher nutrient concentration) can cause coral bleaching (Fabricius et al., 2007; Humphrey et al., 2008; Lirman and Manzello, 2009). Corals respond to stress events by changing their growth rate, losing zooxanthellae, varying their fecundity, reducing planula larval survival, and altering their metabolism (Brown and Howard, 1985). For example, studies have reported that high levels of dissolved inorganic nitrogen and phosphorus could significantly change the physiology of stony corals, including reduced calcification, increased zooxanthellae concentration, and potentially higher rate of coral diseases (Marubini and Davies, 1996; Bruno et al., 2003). Investigations of the coral reefs in the northeast of Brazil (Costa et al., 2000) and in Kenya (Lambo and Ormond, 2006) indicate that the rising turbidity from land-derived input is the main factor culpable for the high bleaching rate and the low recovery degree of coral. Xing et al. (2012) showed that corals with higher density of zooxanthella exhibit stronger tolerance and anti-albinism in turbid water. In the three worldwide coral thermal bleaching events, i.e., in 1997–1998, 2010, and 2015–2016 (Hughes et al., 2018), Acropora and Pocillopora spp. (branching coral) appeared susceptible, while *Porites* and *Goniopora* spp. appeared resistant to environmental stress. Other studies also suggested that different types of reef-building corals had varying tolerance to water turbidity (Xing et al., 2012; Li et al., 2015), and massive corals (e.g., *Galaxea fascicularis* and *Porites lutea*) appeared relatively more tolerant than branched corals (e.g., *Acropora millepora*, *A. nasuta*, and *Pocillopora damicornis*). Furthermore, each coral species responded differently to stress depending on their own shape, scope of the stress tolerance, resistance to pressure, ability to regenerate after disturbance, as well as the associated *Symbiodiniaceae* (Buddemeier and Fautin, 1993; Fabricius, 2005).

Generally, key factors for corals on their resilience against and recovery from bleaching include the level of energy reserve, the shift in endosymbiont type, and the heterotrophic plasticity (Schoepf et al., 2015; Castrillón-Cifuentes et al., 2017). Grottoli et al. (2014) reported that *Porites astreoides* is more sensitive to bleaching than *Orbicella faveolata* and *Porites divaricata* because it has a relatively inflexible assemble of *Symbiodiniaceae* with different genetic types and also a much lower energy reserve baseline than the others. *G. fascicularis* was found to have a complex symbiotic system (Blackall et al., 2015), like flexible symbiotic systems with both clades C and D at regional scales (Huang et al., 2011) and local scales (Zhou et al., 2012) in the South China Sea. Besides, the heat tolerant *Symbiodiniaceae* clade D1a was observed in corals after bleaching events in the Pacific and the Caribbean (Baker et al., 2004; Lajeunesse et al., 2009; Keshavmurthy et al., 2012). Heterotrophy can be crucial in maintaining the physiological functions of corals when autotrophy is depressed. For example, Rodrigues and Grottoli (2006, 2007) studied the change of physiological indicators in the recovery of bleached corals and found dynamic change in trophic status of *Montipora capitata*, and found that photoautotrophy was predominant before bleaching and after recovery but heterotrophy was the major energy source in early recovery. Both heterotrophic ability and energy reserve were found to have a significant impact on the resilience of bleached corals from the studies of *Porites compressa, P. lobata*, and *M. capitata* (branching form) in Kaneohe Bay, Hawaii (Grottoli et al., 2006). Nevertheless, Schoepf et al. (2015) demonstrated that for *P. divaricata*, *P. astreoides*, and *O. faveolata* suffering from annual bleaching, rapid recovery relies less on heterotrophic source but more on the level of energy reserve. These findings indicate that coral species with high carbon retention capability have an advantage in recovering from bleaching.

Corals in the South China Sea have experienced bleaching due to environment perturbation, e.g., global warming, typhoon, flood, coastal pollution, *etc.* (Yu, 2012). Mild to moderate bleaching events have been documented in the South China Sea, including Dongsha (Tkachenko and Soong, 2017), the southern Hainan Island (Li et al., 2012), Taiwan (Kuo et al., 2012), Hong Kong (Xie et al., 2017), *etc.* When effective mechanisms for stress resistance are lacking, regional mortality of corals may rise severely (Ladner et al., 2012). Research interest is increasing on understanding how stress decreases the resistance of corals to perturbation and how acclimatization enhances the resilience of corals against future stress (Castrillón-Cifuentes et al., 2017). Nevertheless, for corals about the Hainan Island of the South China Sea, little has been studied on the effects of chronic negative environment on their physiology, adaptive strategies, and acclimatization. More research is needed to reveal the physiological and biochemical characteristics of corals and their physiological responses and coping strategies to environmental changes, to thus help understand which coral species will become dominant as a result of natural selection and then provide theoretical guidelines for the conservation and restoration efforts in managing the coral reef ecosystems (Hughes et al., 2013; Li et al., 2015).

In 2018, we collected the data of the water environment and the coral reef communities about the Wuzhizhou Island (WZZ) in South China Sea. In this work, we studied the physiological changes of four dominant reef-building corals determined in the prior work, including *Galaxea fascicularis*, *Pocillopora verrucosa*, *Montipora truncata*, and *Porites lutea*. We used both physiological and biochemical methods to assess the long-term acclimatization of these four corals species at WZZ under chronic environmental disturbance. From these reef-building corals we evaluated (i) how do the coral holobiont adapt to perennial environment stress, (ii) which traits are associated with long-term acclimatization, and (iii) what are the intrinsic metabolic strategies of different corals under external environmental stress.

## Materials and Methods

### Study Site and Sample Collection

**FIGURE 1:**
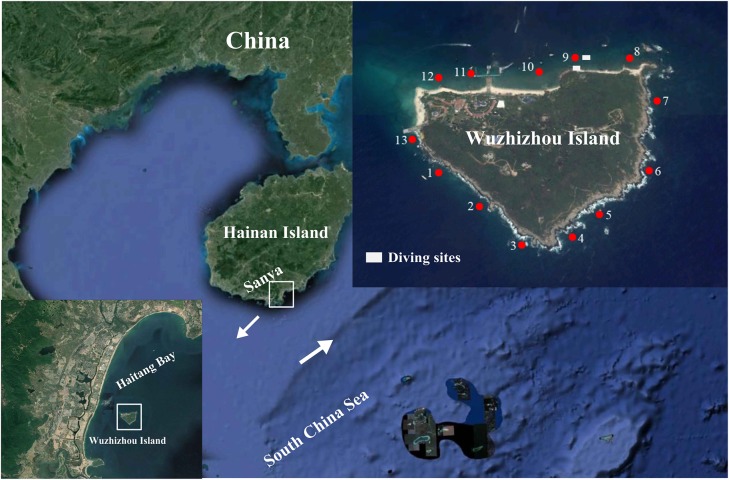
Three sampling sites (3, 4, and 9) in coral reefs of the Wuzhizhou Island. Figure 1 is modified with permission from Li et al. (2019). The 13 study sites are divided by cluster analysis into the south zone (1–7) and the north zone (8–13) based on the benthic composition of the coral reefs (Li et al., 2019).

The WZZ Island (109°45′ E, 18°18′ N) is located off the southeastern coast of the Hainan Island in South China Sea. It is affected by northeast wind and waves in the winter and southerly wind and waves in the summer (Zhang et al., 2006). The 13 study sites are divided by cluster analysis into the south zone (1–7) and the north zone (8–13) based on the benthic composition of the coral reefs (Li et al., 2019). The northern zone has a sandy coastline while the southern zone has a rocky coastline, and the coastline pattern may have a strong impact on the reef development at WZZ (Li et al., 2019). In July 2018, coral samples were collected at the depth of 4–6 m from the northern zone (site 9 in Figure 1) and southern zone (sites 3 and 4 in Figure 1) of WZZ. Coral samples were collected with a chisel from four to six healthy coral colonies that were separated by at least 5 m. Small branches of corals were collected for branching coral communities, and massive corals were chipped into pieces of about 20 cm^2^ in size. All samples were placed in individual plastic bags filled with seawater. The partial coral pieces were immediately washed with filtered seawater, preserved in 95% ethanol on board, transported to the lab without delay, and stored at −20°C in a refrigerator until use.

### Environmental Data

All parameters were measured three times. Steady readings of temperature were measured at the sampling area with HOBO loggers (Tempcon Inc., United States). The seawater salinity and pH were recorded *in situ* with a portable pH/conductivity meter (420C-01A Orion Star, Thermo Fisher Scientific, United States). Turbidity was assessed using an AQUAlogger 210 (Aquatec, United Kingdom). Effective underwater light intensity was determined with a LI-COR (LI-192SA, United States) underwater photo quantum measurement recorder. Samples of bottom level water were collected (100 mL) and filtered (Whatman GF/F, Φ47 mm, United Kingdom) to analyze the composition of inorganic nutrients on a Skalar SAN^plus^ auto-analyzer (Skalar, Netherlands). The dissolved nutrients (DIN) is the sum of ammonium salt, nitrite and nitrate.

### *In situ* Photobiological Variables

A mini-PAM fluorometer (Walz, Germany) was used to determine the photosynthetic variables of corals. This instrument can measure the rapid light curves (RLCs) and the relative electron transport rate (ETR, μmol electron m^–2^s^–1^) for photosystem II with previously described settings (Schreiber et al., 1986; Hennige et al., 2008). Data were fitted to the Jassby and Platt (1976) model, which describes the dependency of ETR on the incident light intensity (E), by least squares non-linear regression to determine the light use efficiency (i.e., light-limited) α (mol electron per mol photon), the maximum relative electron transport rate (i.e., light-saturated) rETR_max_ (μmol electron m^–2^s^–1^), and the minimum saturating irradiance (E_k_), respectively (Suggett et al., 2012). After the photosynthetic variables of corals were measured, coral samples were pretreated and cryogenically transported to the laboratory for subsequent physiological and biochemical determinations.

### Physiological Parameters

All parameters were measured for three times. Coral samples were collected in a plastic bag after photobiological measurements. Tissues were removed from the skeleton with a Waterpik flosser (WP-70EC, Water pik, Inc.) containing filtered seawater (0.45 μm, Whatman, United Kingdom), and tissue biomass was then measured (Fitt et al., 2000). The surface area of corals was determined by the aluminum foil method (Li et al., 2006), and the ratio between the ash-free dry weight and the surface area was calculated to give the tissue biomass. Chlorophyll a (Chl a) from coral samples was extracted with 100% acetone in darkness and determined with a spectrophotometer (Lei et al., 2008). The density of symbiotic algae was assessed according to the method of Li et al. (2006).

Carbohydrates were measured as previously described (Dubois et al., 1956). Total lipid was determined according to the method of Grottoli et al. (2004) with modification. The coral samples (wet weight about 2 g) were extracted with 36 ml of CM solution (V_methanol_:V_chloroform_ = 1:2), add 1/5 of solution volume (7 ml) of 0.88% KCl, followed by dark treatment for extraction about 24 h, then the extract was placed in the 39°C N_2_ gas vacuum environment to evaporate and dry. Total protein was measured by the bicinchoninic acid method (Smith et al., 1985) with a Modified BCA Protein Assay Kit (Sangon Biotech, China). Total energy reserves were calculated as the sum of total lipids, carbohydrates, and proteins. The SOD (superoxide dismutase) enzyme was measured with an Activity Kit (Nanjing Jiancheng Bio-Engineering Institute Co., Ltd.).

### DNA Extraction and Amplicon Sequencing

Total DNA was extracted according to the method given in Zhou and Huang (2011). The DNA Extraction Kit for Marine Animal Genome (TIANGEN, Beijing, China) was used. The extracted DNA samples were used as PCR templates after quality check and purity filter. The primers of ITSintfor2 and ITS2clamp (F: 5′-GAATTGCAGAACTCCGTG-3′; R: 5′-CGCCCGCCGCGCCCCGCGCCCGTCCCGCCGCCCCCGCC CGGGATCCATATGCTTAAGTTCAGCGGGT-3′) (LaJeunesse, 2002) were used to produce PCR amplification of the *Symbiodiniaceae* ITS2 region of the rDNA. The 330–360 bp ITS2 fragments were purified using the SanPrep Column DNA Gel Extraction Kit (Sangon Biotech, China). All amplified PCR products were then sequenced with a paired-end (PE) 300 bp × 2 strategy on a ABI3730 sequencer operated by Sangon Biotech (Shanghai, China). DNA sequences were identified in GenBank by BLAST and by phylogenetic analysis (Zhou and Huang, 2011). Data were submitted to the GenBank under accession number MN630169-MN630173. The ITS2 sequences generated in this study and previous studies (LaJeunesse et al., 2005; Granados-Cifuentes and Rodriguez-Lanetty, 2011; Zhou et al., 2012; Kavousi et al., 2015; Ng and Ang, 2016; Wong et al., 2016) were aligned using CLUSTAL W version 1.8. A phylogenetic tree of ITS2 was constructed using the neighbor-joining algorithms within the MEGA version 7.0 (Kumar et al., 2016). A bootstrap resampling was carried out for 1,000 replicates to assess relative branch support.

### Stable Isotopic Analysis

Samples for carbon isotope analysis were prepared according to the method given in Rodrigues and Grottoli (2006). The zooxanthellae and coral tissue were separated and washed by repeated centrifugation at different speeds before collection. The δ^13^C values were finally measured on a Delta Plus XP Isotope Ratio Mass Spectrometer at the Guangzhou Institute of Geochemistry, Chinese Academy of Sciences (Guangzhou, China). The relative contribution of autotrophy versus heterotrophy for corals was determined by calculating Δ = δ^13^C_h_ −δ^13^C_z_, that is, subtracting the carbon isotopic value of the host tissue (δ^13^C_h_) by that of the zooxanthellae (δ^13^C_z_) (Muscatine et al., 1989). Compared with photosynthesis, heterotrophy contributes little to the fixed carbon pool when δ^13^C_h_ is greater than δ^13^Cz, but contributes more when δ^13^C_h_ is less than δ^13^Cz. Therefore, the relative contribution of heterotrophy to the fixed carbon of the coral increases when Δ is lower (Rodrigues and Grottoli, 2006; Schoepf et al., 2015).

### Data Analysis

All experiments were repeated at least three times. Statistical analysis was carried out using the statistical package SPSS 17.0 for Windows (SPSS Inc., Chicago, IL, United States). Independent-Samples T Test was adopted to compare data at a significance level of *P* < 0.05. Descriptive statistics were expressed as mean ± standard deviation.

## Results

### Molecular Analysis of Zooxanthellae Clades and Types

**FIGURE 2:**
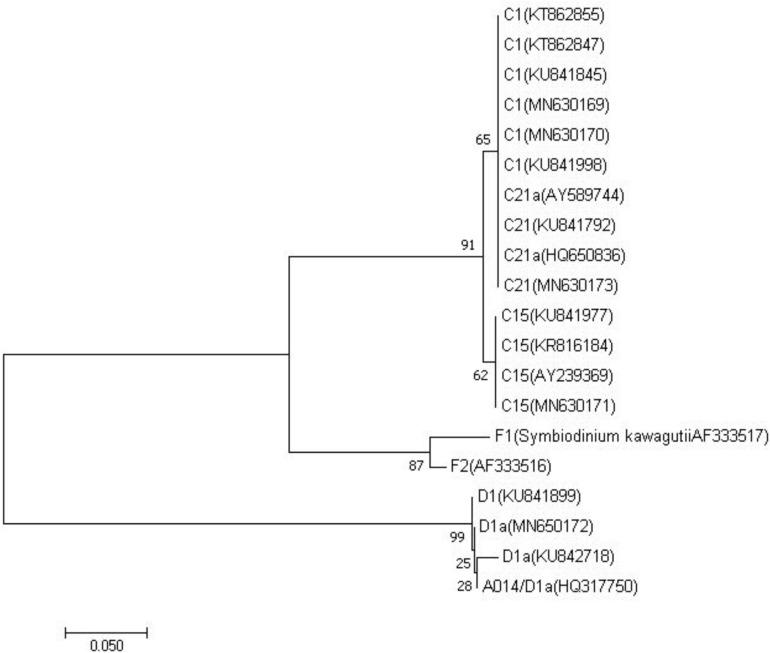
Phylogenetic reconstruction of internal transcribed spacer 2 genes from different symbionts within clade C and D detected in the reef building corals from this study and previous studies (LaJeunesse et al., 2005; Granados-Cifuentes and Rodriguez-Lanetty, 2011; Seyfabadi et al., 2011; Zhou et al., 2012; Arif et al., 2014; Kavousi et al., 2015; Ng and Ang, 2016; Wong et al., 2016) using neighbor joining algorithms. Sister lineages to clade C represented by F1 (Symbiodinium kawagutii AF333517) and F2 (AF333516) in clade F were used as outgroups. Corresponding GenBank accession numbers are provided next to each symbiont type.

PCR amplification of ITS2 from zooxanthella of four host species produced a single amplicon of approximate 330-360 base pair (Supplementary Figure S1). All sequences of zooxanthella in this study were identified based on phylogenetic analysis, covering clades C and D (Figure 2). Four subfamilies were identified, namely C1, C15, C21, and D1a (Supplementary Table S1, Supporting Information). Among them, *M. truncata* and *P. verrucosa* were mainly associated with the zooxanthella type C1, while *P. lutea* was mainly associated with the zooxanthella type C15. Interestingly, *G. fascicularis* was mainly symbiotic with the zooxanthella type C21 in the north (site 9) but with zooxanthella type D1a in the south (site 4).

### The Variation of Environmental Parameters

Supplementary Table S3 lists the variation of the environmental parameters during the survey time. Turbidity, NO_3_^–^, and seawater temperature were notably higher at site 9 than at sites 3 and 4 (*P* < 0.05). For instance, the average turbidity at site 9 in the north was 3.20 FTU, whereas the average turbidity at sites 3 and 4 in the south was 1.22 FTU, approximately only half as much compared with that of the north. In addition, the seawater temperature was on average 1°C higher in the north than in the south (Supplementary Table S3). No significant difference was noted for the variation of pH across the studied sites. The DIN and salinity were significantly higher at site 9 than at sites 3 and 4 at the depth of 3 m, but not so significant difference at 8 m. On the whole, the DIN value ranged from 4.04 to 8.02 μM.

### Changes in the Photosynthesis of Corals

**FIGURE 3:**
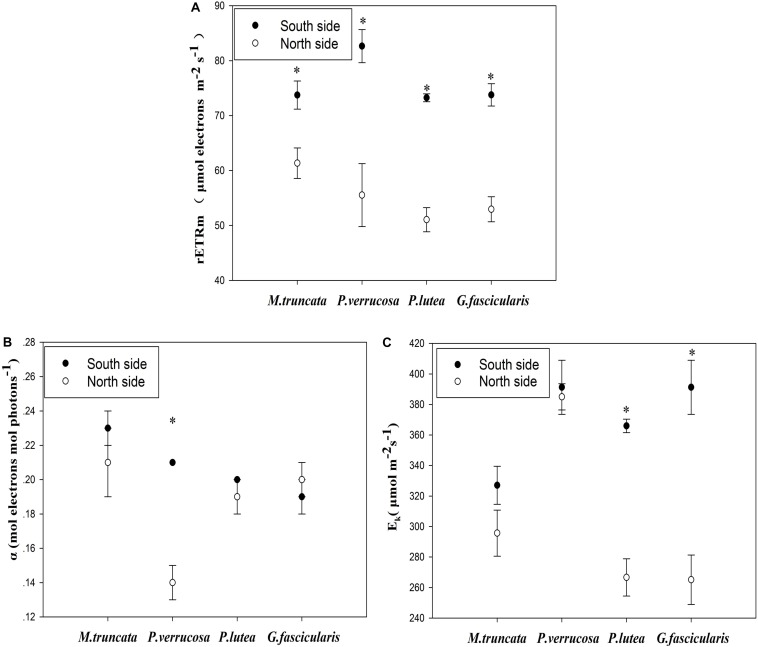
Photosynthesis of corals. **(A)** The maximum relative electron transport rate (rETRmax). **(B)** The light use efficiency (α). **(C)** The minimum saturating irradiance (Ek).

The average maximum relative electron transport rate (rETR_max_) was 55.22 and 75.86 μmol electrons m^–2^ s^–1^ for corals in the north and south side, respectively (Figure 3A). For all coral species, rETR_max_ was significantly lower (*P* < 0.05) in the north than in the south. The light use efficiency (α) of *P. verrucosa* was significantly lower in the north than in the south (Figure 3B), but the light use efficiency of the other three coral species were not distinguishable between the north and the south. The minimum saturating irradiance (E_k_) of *P. lutea* and *G. fascicularis* was significantly lower in the north than in the south (Figure 3C).

### Zooxanthellae Density and Chl a of Corals

**FIGURE 4:**
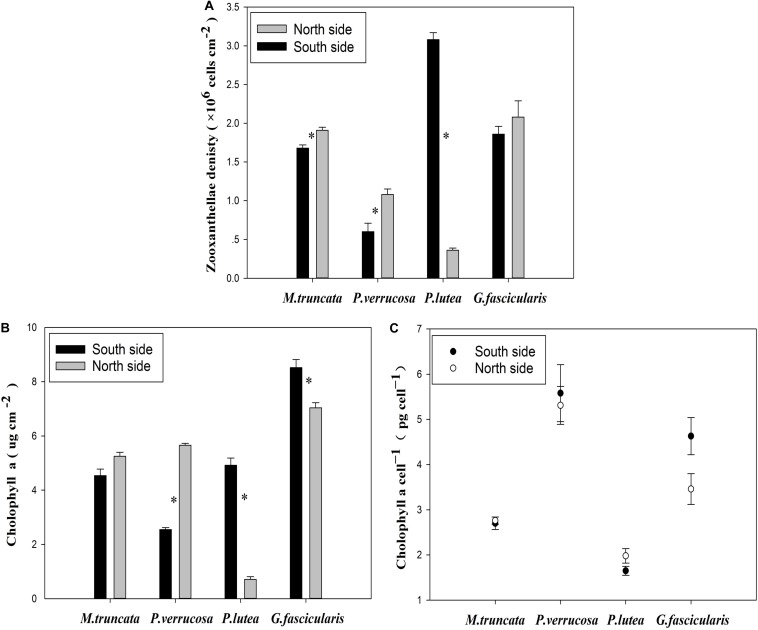
The zooxanthellae density of corals. **(A)** Zooxanthellae density. **(B)** The content of Chl a. **(C)** The Chl a content of each zooxanthellae cell.

The average density of zooxanthella was 1.35 × 10^6^ and 1.81 × 10^6^ cells cm^–2^ for the corals in the north and in the south, respectively (Figure 4A). The density of zooxanthella ranged in 0.36–3.08 × 10^6^ cells cm^–2^. For *P. verrucosa* and *M. truncata*, the density of zooxanthella was significantly higher in the north than in the south, but the reverse was noted for *P. lutea*. No significant difference was found in the density of zooxanthella of *G. fascicularis* between the north and the south.

The Chl a content of corals varied essentially in the same way as the density of zooxanthella. The Chl a content of *G. fascicularis* was significantly lower in the north than in the south (Figure 4B). Besides, the Chl a content of each zooxanthellae cell in *P. lutea* was significantly lower (*P* < 0.05) than that in all three other coral species (Figure 4C).

### Biomass of Corals

**FIGURE 5:**
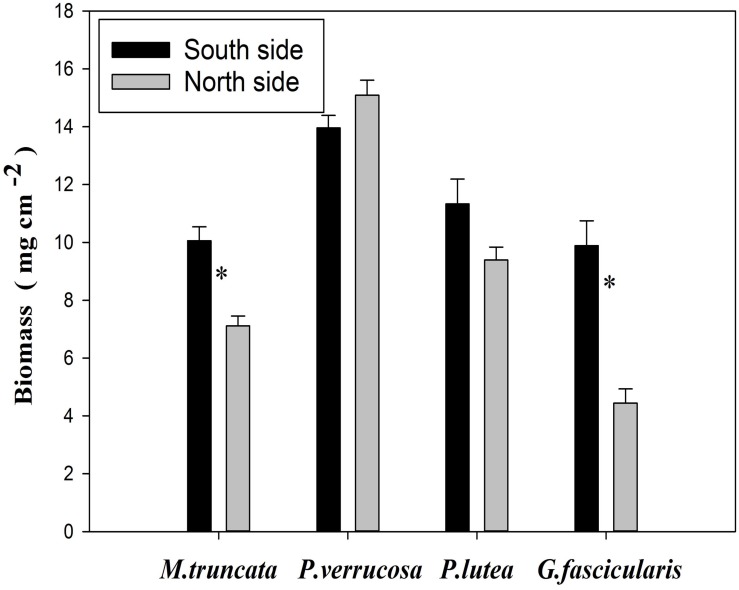
Biomass changes of corals. ^∗^*P* < 0.05.

The average biomass of corals reached 9.01 and 11.31 mg cm^–2^ in the north and in the south, respectively (Figure 5). The values of coral biomass ranged in 4.44–15.09 mg cm^–2^, and the average biomass of *P. verrucosa* was 14.53 mg cm^–2^. The biomass of *G. fascicularis* and *M. truncate* was significantly lower in the north than in the south (*P* < 0.05), but no such difference was found for *P. verrucosa* and *P. lutea*.

### Stable Isotopic C of Corals

For all coral species, the average Δ ranged in 0.58–3.86 mg cm^–2^ (Supplementary Table S2). Specifically, the average Δ was 2.85 and 0.65 mg cm^–2^ for *P. verrucosa* and *G. fascicularis*, respectively. For *P. lutea*, Δ was significantly lower in the north than that in the south (*P* < 0.05), but the reverse was true for *M. truncata*.

### Energy Reserve of Corals

**FIGURE 6:**
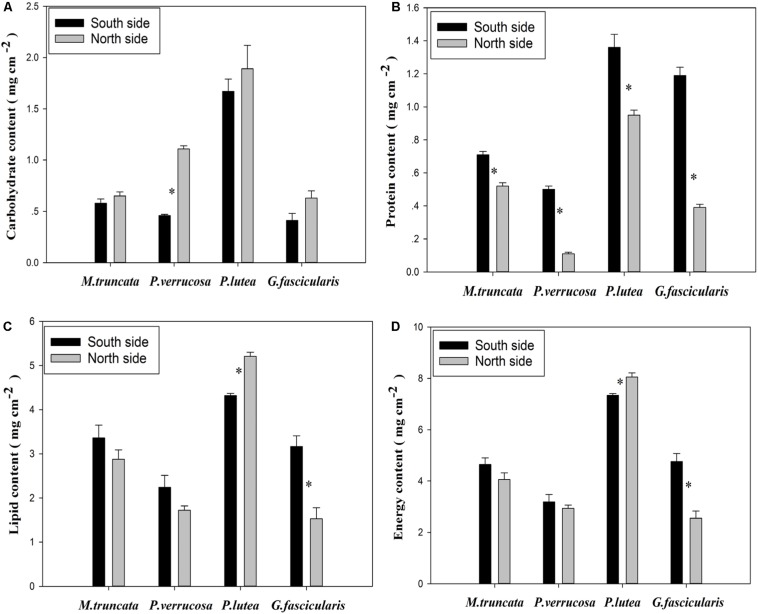
The energy reserve of corals.

As shown in Figure 6A, *P. verrucosa* had notably higher carbohydrate content in the north than in the south, but no such difference could be found for the other three species (*P* < 0.05). The average protein content of the corals was 0.49 and 0.94 mg cm^–2^ in the north and in the south, respectively (Figure 6B), and all four coral species had lower protein content in the north than in the south. In addition, Figure 6C shows that the lipid content of *P. lutea* was significantly higher in the north than in the south (*P* < 0.05), but the reverse was true for *G. fascicularis*.

The average energy reserve of the corals was 4.40 and 4.99 mg cm^–2^ in the north and in the south, respectively (Figure 6D). The average energy reserve fell in the range of 2.55–8.05 mg cm^–2^. Specifically, the average energy reserve was 7.70 and 3.07 mg cm^–2^ for *P. lutea* and *P. verrucosa*, respectively. The average energy reserve was significantly lower in the north than in the south for *G. fascicularis* (*P* < 0.05), but the reverse was true for *P. lutea* (*P* < 0.05). No significant difference was found in the average energy reserve between the north and the south for *M. truncata* and *P. verrucosa*.

### SOD Enzyme Activity of Coral

**FIGURE 7:**
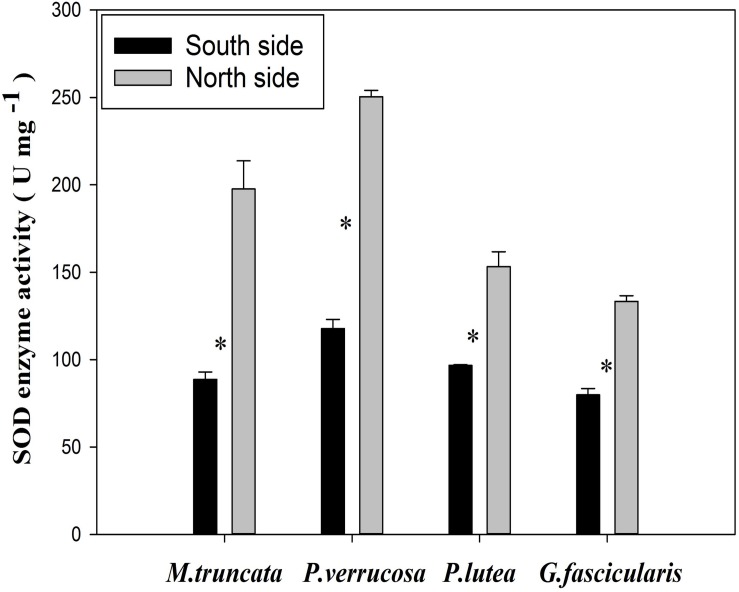
The SOD enzyme activity of corals. ^∗^*P* < 0.05.

The average SOD enzyme activity of the corals was 183.67 and 95.72 U mg^–1^ in the north and in the south, respectively (Figure 7). The SOD enzyme activity of all four corals species fell in the range of 79.82–250.45 U mg^–1^, and was always significantly higher in the north than in the south (*P* < 0.05). In particular, the SOD enzyme activity was more than twice as much in the north than in the south for both *M. truncata* and *P. verrucosa*.

## Discussion

### Physiological Traits and Environment Status

Significant differences were found between the north and the south for the four coral species in their photosynthetic and biochemical traits, including the rETR_max_, biomass, protein content, and the SOD enzyme. The rETR_max_ reflects the maximum potential photosynthetic capacity of an organism in its natural environment, and is an important indicator of the growth state of symbiotic algae (Schreiber et al., 1997; Gao and Zheng, 2010). The results clearly showed that the rETR_max_ of the four coral species was significantly lower in the north than in the south. Down-regulation of photosynthesis or damage to the photosystem has been previously reported for the northern coral at the Florida Key Largo National Marine Sanctuary (Warner et al., 1999). In fact, impaired photosynthetic function of symbionts is one of the first consequences of thermal stress (Warner et al., 1999; Fitt et al., 2009).

Previous studies also found that exogenous antioxidant enzymes (e.g., SOD) may play an important role in suppressing reoxygenation injury (Mccord, 1985; Bhagooli and Hidaka, 2004a) for photosynthetic organisms suffering from hyperoxia, high temperature, or UV radiation (Valenzeno and Pooler, 1987; Lesser et al., 1990). The current results found that the SOD enzyme activity of the four coral species were significantly higher in the north than in the south. Bhagooli and Hidaka (2004a) demonstrated the defensive mechanism of corals that induces the synthesis of antioxidant enzymes upon oxidative stress. Thus, the higher SOD of the stony corals in the north indicates stronger stress response. The change in SOD activity has been taken as an early warning for the variation in the concentration of nutrients in the seawater hosting the coral reef (Main et al., 2010). Detrimental variation of environmental parameters such as light intensity, turbidity, and temperature will generate reactive oxygen that are harmful to biological organisms and trigger stress response in corals (Aguilera et al., 2010; Lesser, 2011). Presumably, the corals suffer from higher environmental stress in the north side of WZZ than in the south, such as high nutrient concentration, temperature perturbation, and turbidity (changes in sedimentation and suspending particles). Indeed, turbidity, nitrate concentration (NO_3_^–^), and seawater temperature were significantly higher in summer of 2018 at site 9 (Supplementary Table S3), all of which contributed to greater disturbances to the corals. The results were consistent with previous reports that showed heavier environmental stress in the north side of WZZ because of tourism development and human activities (yearly mean values of four seasons, temperature: 27.10 and 26.54°C, turbidity: 0.41 and 0.28 FTU, DIN: 3.37 and 2.87 μM for north and south side of WZZ, Li et al., 2019).

### Environmental Impact on Symbiotic Relationship

The healthy growth of corals relies on the relationship between reef-building corals and the symbiotic dinoflagellates (zooxanthellae). Clade C zooxanthella is widely distributed in low-latitude tropical corals (Rodriguez-Lanetty et al., 2001; Lajeunesse et al., 2008). The current results clearly showed a dominating presence of clade C (Supplementary Table S1) zooxanthella at all sampled sites. However, *G. fascicularis* was mainly symbiotic with either zooxanthella C21 or D1a because *G. fascicularis* can be flexibly symbiotic with either clade C or D according to local conditions. It has been found in the waters of Singapore that symbiosis between coral and clade D decreases with rising turbidity (Cooper et al., 2011). Studies have shown that many clade C zooxanthella members are more beneficial to promoting coral growth and calcification than other zooxanthella (Mieog et al., 2009; Jones and Berkelmans, 2010). In the Indo-Pacific region, clades C and D have been reported as the dominant types of zooxanthella for corals (Baker, 2003; LaJeunesse, 2005). Besides, it has been hypothesized that corals can adapt to environmental perturbations by shifting from existing symbionts and switching to novel symbionts (Buddemeier and Fautin, 1993; Baker, 2003; Baker et al., 2004). Our results suggested that *G. fascicularis* shifted its primary zooxanthella because of local environmental stress at site 9 of WZZ, *i.e.*, from both chronic high turbidity and rising temperature.

The four coral species adopted significantly different photosynthetic physiology of symobiotic zooxanthella in response to the environmental stress in the north region of WZZ. In particular, the density of C1 zooxanthellae from *M. truncata* and *P. verrucosa* was significantly higher in the north than in the south, and the C1 zooxanthellae from *P. verrucosa* in the north had significantly lower light use efficiency. It has been proposed that elevated temperatures and UV radiation can cause photosynthesis suppression when symbiotic dinoflagellates suffer from chronic or dynamic photoinhibition (Lesser, 1996; Bhagooli and Hidaka, 2004a, b). Moreover, higher turbidity also reduces the amount of light available for photosynthesis (Pollock et al., 2014). Xing et al. (2012) showed that corals with higher density of zooxanthella exhibit stronger tolerance and anti-albinism in turbid water. Since the minimum saturating irradiance for *M. truncata* and *P. verrucosa* were the same in the north compared with in the south, the photosynthetic tolerance of *M. truncata* and *P. verrucosa* must be strong. It is possible that the zooxanthella of *M. truncata* and *P. verrucosa* in the north reversibly regulate photosynthesis between periods of high radiation and high turbidity. The decreased efficiency, as was observed, is the trade-off between protection and damage, by which extra energy can dissipate in the form of heat or through the conversion of key proteins (especially D1) of the photosystem II (PSII) (Chow, 1994; Osmond, 1994). In the case of high turbidity, sediment deposition covers corals and requires energy to be removed through energy budget allocation (Pollock et al., 2014), and *M. truncata* and *P. verrucosa* in the north might have increased their density of zooxanthella as a coping mechanism (Warner et al., 1999; Salih et al., 2000). The density of zooxanthellae and the Chl a content of *M. truncata* and *P. verrucosa* were indeed higher in the north than in the south. Thus, *M. truncata* and *P. verrucosa* can maintain adequate autotrophic nutrient input for growth and energy supply under the negative environment.

However, no difference was found between the north and the south in the density of zooxanthella for *G. fascicularis*, and the density of zooxanthella was significantly lower in the north than in the south for *P. lutea*. Higher sea surface temperature and photosynthetic radiation have been suggested to reduce symbiont density for some shallow-water corals (Warner et al., 1996, 1999; Brown et al., 2000). Since the minimum saturating irradiance of *P. lutea* and *G. fascicularis* was significantly reduced in the north of WZZ, the photosynthetic tolerance of *P. lutea* and *G. fascicularis* must be poor under such unfavorable environment. Some visible or UV radiations can generate oxygen radicals via photodynamic production to damage the photosystem II at the D1 protein (Valenzeno and Pooler, 1987; Richter et al., 1990; Tschiersch and Ohmann, 1993). Thus, the zooxanthella of *P. lutea* and *G. fascicularis* in the north of WZZ might have suffered from irreversible photosystem damage during periods of high radiation. Besides, *P. lutea* showed lower Chl a content of single zooxanthella cell than the other three coral species. Although the *Porites* sp. with thick tissues have shown resistance in several bleaching events (Bruno et al., 2001; Aeby et al., 2003; De’ath et al., 2012), *P. lutea* in the north side of WZZ largely released the weak C15 zooxanthellae to reduce load consumption, possibly because the symbiotic decomposition depends on the degree of stress and involves both the zooxanthella and the host (Bhagooli and Hidaka, 2004b). The collaboration between a robust host equipped with good photo-protection capacity and a robust zooxanthella with photo-physiological tolerance will constitute the best resistance to stress. That is, enhanced resistance of the zooxanthellae and/or the host can surely improve the robustness of corals against environmental disturbance (Keshavmurthy et al., 2012, 2014). Therefore, the robust host of *P. lutea* might release the weak zooxanthellae and/or combine with other robust zooxanthellae to adapt to the unfavorable environment in the north of WZZ. As a result, both the Chl a content and the density of zooxanthella for *P. lutea* were significantly lower in the north than in the south.

### Heterotrophic Energy Contribution

The host in corals may change its trophic mode to enhance resistance and sustain a positive energy balance in an environment with long-term disturbance (Anthony and Larcombe, 2000; Schoepf et al., 2015). When corals suffer from environmental stress and lose their zooxanthellae, phototrophic input attenuates and heterotrophy sometimes becomes the primary source of nutrients (Sebens et al., 1996; Houlbrèque and Ferrierpagès, 2010). The interaction between light intensity and other environmental factors (e.g., temperature, Franklin, 1994; turbidity, Anthony and Fabricius, 2000) may cause a shift in the balance between protection and damage. We measured Δ = δ^13^C_h_ −δ^13^C_z_ to evaluate the heterotrophy of the studied corals. It was found that Δ was significantly higher for *P. lutea* in the north than in the south, which corresponded to a higher contribution of heterotrophic intake in the north where the environment was adverse. In the case of *G. fascicularis*, the Δ value did not change significantly from the south to the north, indicating that the host in the north still keeps high autotrophic ability. Leal et al. (2016) found that the autotrophic intake is firstly used for the respiration consumption of the symbiont when the autotrophy of corals is limited, and the energy needed for coral growth depends mainly on heterotrophy. Therefore, by increasing the ingesting rate of heterotrophic food (e.g., suspended particulate matter, SPM), *P. lutea* can compensate for lower phototrophic input at higher turbidity to thus alleviate stress and sustain energy input.

### Energy Balance and Budget

Lipids, proteins, and carbohydrates are the main energy substances of reef building corals, and they readily reflect changes in coral health (Yellowlees et al., 2008; Lesser, 2013). The synthesized carbon surplus from biological functions is the main energy source for calcification and reproduction (Castrillón-Cifuentes et al., 2017). According to our energy survey, the four identified coral species all had significantly lower protein content in the north compared with in the south, showing an adjusted energy structure. Thanks to sufficient autotrophic input after a dramatic increase in the number of zooxanthella, *M. truncata* and *P. verrucosa* had similar energy reserve in the north and in the south, but they had different energy structure. For instance, the carbohydrate content of *P. verrucosa* significantly increased in the north than that in the south. Besides, *P. lutea* seems to maintain a higher energy reserve than other coral species even though its autotrophic input is limited, probably because *P. lutea* has higher baseline energy reserve and/or the energy budget is balanced by subtraction. Grottoli et al. (2014) and Schoepf et al. (2015) showed that *Porites astreoides* keeps a high level of energy reserve in response to long-term bleaching stress, which is essential for a rapid recovery. Besides, during stressful periods, trade-off is made to maintain crucial functions by compromising obvious scope to growth (Zamer and Shick, 1987; Grottoli et al., 2014) or aberrant fecundity (Patton et al., 1977; Castrillón-Cifuentes et al., 2017), and energy budget is balanced by subtraction (Edmunds and Davies, 1989). For *G. fascicularis*, although it maintains high heterotrophic ability, its energy reserve was notably lower in the north compared with in the south side of WZZ. Therefore, its survival in the north of WZZ might be threatened in the future. The current results demonstrated different adaptive strategies of the coral species under environmental stress.

### Coral Community Change

The coral coverage and the juvenile coral density in the north of WZZ were significantly lower than in the south, and at a water depth of 8 m, the diversity of coral species was significantly lower in the north than in the south (Li et al., 2019). The predominant coral communities were *Proties*, *Montipora*, *Acropora* and *Pocillopora* in the north, and *Montipora*, *Galaxea*, *Acropora*, and *Pocillopora* in the south (Li et al., 2019), probably because these species differ in their environmental tolerance and resistance to bleaching. For example, in 2003, coral bleaching mainly occurred in *Montipora* and *Pocillopora* corals, whereas the *Porties* corals that suffered from massive bleaching in the Hawaii islands were few (Aeby, 2002). In general, branching *Acropora* and *Pocillopora* are more susceptible to thermal stress than other massive growth forms of corals. Interestingly, the turbid shallow reef communities at Pulau Satumu of Singapore exhibited an unorthodox pattern of bleaching susceptibility in July 2010 (Guest et al., 2016). The unusual pattern may be ascribed to several factors, including symbiotic relationship, turbidity, and heterotrophy, prior acclimatization, all of which contributed to the high overall resistance of corals to acute thermal stress at the observed site. Some corals that have been classified as losers under climate change may be more resilient and/or adaptive than previously expected (Grottoli et al., 2014). If environmental disturbance is too stressful for the corals to adapt, changes may occur in the coral community such that species with phenotypic plasticity or genetic adaptation will gradually dominate (Bell, 2013). However, the dominant coral species is selected by a variety of natural factors according to local conditions and is therefore not immutable.

## Conclusion

Corals at the waters about the WZZ suffer from greater environmental stress in the north than in the south, including stronger radiation, higher turbidity, and more suspended particles. As a result, significant differences were found for the four dominant coral species in their physiological and biochemical characteristics, including rETR_m__ax_, SOD, and protein content, between the north and the south. The physiological states were healthier in the south than in the north, and matched the spatial variation of the coral communities.

The four dominant reef-building corals in the north responded to complex environmental stress and showed different adaptation strategies. *M. truncata* and *P. verrucosa* increase the density of symbiotic zooxanthella to balance the decline of photosynthetic efficiency and supply energy. *P. lutea* releases a number of zooxanthellaes to improve heterotrophic feeding, and/or change the energy budget mode to maintain high energy reserve. *G. fascicularis* alters its primary symbiotic zooxanthella type and hosts to maintain high heterotrophy. These corals maintain their presence by adopting their own successful resistance mechanisms at symbiotic relationship and energy metabolism level over time against long-term disturbances such as elevated turbidity or temperature.

## Data Availability Statement

These DNA data were submitted to the GenBank under accession number MN630169–MN630173.

## Ethics Statement

All animal-involving experiments of this study were approved by the Ethics Committee of Hainan University and local government.

## Author Contributions

HX and XL wrote the manuscript. HX, BF, MX, YR, JX, and YZ performed the research. HX analyzed the data. HX, XL, and AW designed the research. All authors reviewed the manuscript.

## Conflict of Interest

The authors declare that the research was conducted in the absence of any commercial or financial relationships that could be construed as a potential conflict of interest.
