# Supplementary material for: Physiological Characteristics and Environment Adaptability of Reef-Building Corals at the Wuzhizhou Island of South China Sea
Source: Front Physiol. 2020 Apr 29;11:390. doi: 10.3389/fphys.2020.00390 (PMC7201098; doi:10.3389/fphys.2020.00390)
Supplement: Supplementary file 1 [file Table_1.pdf]

Supplementary Material

Figure S1. This is a figure with sub figures, (A) agarose gel electrophoresis of genome DNA and (B) PCR products of ITS2 gene for zooxanthellae.

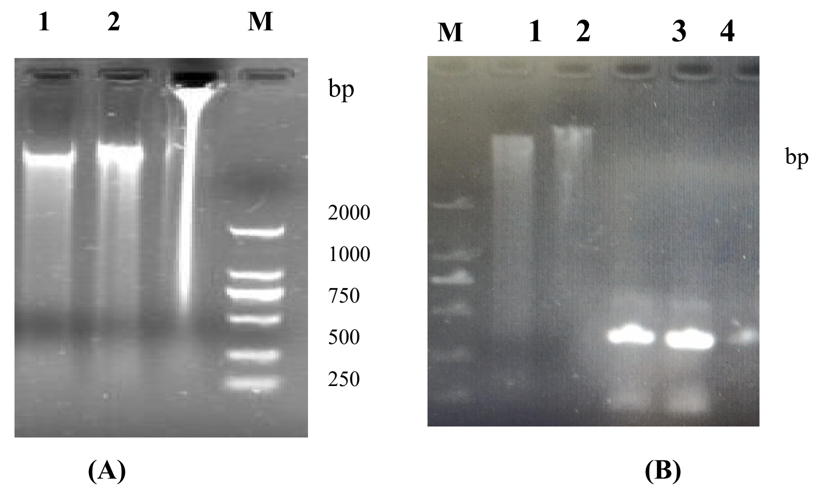

Note: Lanes 1-2 are genome DNA; lanes 3-4 are ITS2 PCR products; M, DNA ladder.

Table S1. DNA genes for Zooxanthellae in the coral.

Coral genues	Sequence ID	Length (bp)	Sample name	Gene types	Sampling coral habitats	Site and Depth
Galaxea fascicularis	MN630172	381	4#6-GF	D1a	Wuzhizhou Island	#4, 6m

	MN630173	360	9#4-GF	C21	Wuzhizhou Island	#9, 4m
Pocillopora verrucosa	MN630170	332	3#6-PV	C1	Wuzhizhou Island	#3, 6m
			9#4-PV	C1	Wuzhizhou Island	#9, 4m
Montipora truncata	MN630169	334	3#6-MT	C1	Wuzhizhou Island	#3, 6m
			9#4-MT	C1	Wuzhizhou Island	#9, 4m
Porites lutea	MN630171	302	4#8-PL	C15	Wuzhizhou Island	#4, 8m
			9#6-PL	C15	Wuzhizhou Island	#9, 6m

Note: The first and second numbers in the sample name indicate site and depth respectively. Sequences have been deposited in GenBank (accession numbers: MN630169-MN630173.)

Table S2. Stable isotopic C analysis of different corals.

**Coral genus**	**Sample name**	**$\Delta=\delta^{13}C_h - \delta^{13}C_z$**	**Mean ± SE**
Galaxea fascicularis	4#6-GF	0.49–0.67	0.58 ± 0.08
	9#4-GF	0.60–0.84	0.72 ± 0.10
Pocillopora verrucosa	3#6-PV	1.76–2.18	1.97 ± 0.17
	9#4-PV	2.06–2.90	2.48 ± 0.34
Montipora truncata	3#6-MT	0.71–0.83	0.77 ± 0.05
	9#4-MT	2.14–2.18	2.16 ± 0.02*
Porites lutea	4#8-PL	3.35–4.38	3.86 ± 0.42
	9#6-PL	1.72–1.97	1.84 ± 0.01*

Note: The first and second numbers in the sample name indicate site and depth (m) respectively. Stronger heterotrophy is indicated by a smaller value of Δ. "*"

indicates P<0.05.

Table S3. Environmental parameters (mean ± SE) of the sampling sites in the summer.

**Depth**	**Site**	**Temperature**	**pH**	**Salinity**	**Turbidity**	**DIN**	**NH_4^+**	**NO_3^-**	**NO_2^-**
3 m	#3	25.6±0.03	8.15±0.01	33.93±0.01	1.26±0.02	5.19±0.05*	4.44±0.03	0.67±0.02	0.08±0.00
	#4	25.8±0.02	8.17±0.01	34.15±0.02	1.25±0.01	4.85±0.01	4.43±0.10	0.35±0.01	0.08±0.00
	#9	26.8±0.03*	8.17±0.01	31.55±0.01*	3.12±0.10*	7.02±1.42	4.05±1.48	2.86±0.00*	0.12±0.06
8 m	#3	23.7±0.03	8.18±0.00	34.24±0.03	1.21±0.05	4.70±0.10	4.36±0.10	0.26±0.00	0.08±0.00
	#4	23.8±0.02	8.17±0.00	34.27±0.01	1.16±0.02	4.99 ±0.03	4.79±0.01	0.13±0.03	0.07±0.01
	#9	24.8±0.02*	8.16±0.00	34.08±0.01	3.28±0.06*	4.05±0.17	3.45±0.17	0.55±0.02*	0.05±0.02

Note: “*” indicates P<0.05.
